# Donor Blood Management: A Complementary Framework to Patient Blood Management

**DOI:** 10.1002/jha2.70401

**Published:** 2026-09-28

**Authors:** Allameddine Allameddine, Andrea Piccin

**Affiliations:** ^1^ Northern Ireland Blood Transfusion Service Belfast UK; ^2^ Royal College of Surgeons Dublin Ireland; ^3^ Mater Private Hospital Dublin Ireland

**Keywords:** blood inventory management, blood stewardship, blood transfusion, donor advocacy, Donor Blood Management, haemovigilance, Patient Blood Management, transfusion safety

## Abstract

Blood donation is a generous act that saves lives. However, the journey of the donated blood from collection to patient benefit is complex, and donor value may be reduced or lost through collection and processing losses, inventory wastage, expiry, handling errors, inappropriate clinical utilisation and incidents. Patient Blood Management (PBM) has transformed transfusion practice by promoting evidence‐based blood use, yet no equivalent donor‐centred framework exists to protect donor value across the full donation‐to‐transfusion pathway. We propose Donor Blood Management (DBM), built on four interconnected pillars: (i) Collection and processing optimisation, (ii) inventory optimisation, (iii) appropriate utilisation and (iv) haemovigilance and improvement. DBM promotes shared accountability among blood services, hospitals, clinicians and regulators to reduce avoidable losses and maximise the benefit of every donation. We also propose Donor‐to‐Recipient Value Yield (DRVY) as a stewardship metric to measure the proportion of donated blood ultimately transfused appropriately. DRVY is not intended to replace existing vein‐to‐vein data infrastructure but to provide a standardised, quality‐improvement interpretation of linked donor, component and recipient data, complementing PBM within a donor‐centred framework.

**Trial Registration**: The authors have confirmed clinical trial registration is not needed for this submission.

## 1 Introduction

In this paper, donor value refers to the ethical, clinical and operational value created when a donated unit is converted safely and appropriately into patient benefit, while preserving donor health and trust. It is not limited to the physical component itself; it also includes the donor time, willingness, physiological burden and public confidence that make the blood system possible.

Every blood donation is a finite, perishable, and irreplaceable gift: a voluntary act by someone who gives their time and commitment for a patient in need. Stakeholders in transfusion medicine must therefore protect both the safety and quality of blood components and the donor and their gift. These responsibilities are addressed partly through established systems. GMP governs donor qualification, collection, testing, processing and storage, thereby protecting component safety and quality. PBM focuses on patient‐centred care, including whether transfusion is clinically indicated. However, neither framework explicitly follows the donor gift across the entire pathway to ask whether avoidable loss has been minimised and whether the donor burden has translated into appropriate patient benefit.

DBM complements PBM and GMP by using system‐wide data to ask whether blood systems protect donor health, minimise avoidable donor burden, preserve component value and translate donations into appropriate patient benefit.

Clinical appropriateness remains the responsibility of PBM and the treating team, whose focus is appropriate clinical decision‐making and reducing unnecessary transfusion [1, 2, 3, 4]. DBM addresses what happens before that decision point: whether the donor gift has been protected, avoidable upstream losses have been minimised and system‐wide data are used to ensure that donation is translated into patient benefit.

DBM is needed because donor burden and donor value are managed in different parts of the system. Donor eligibility standards protect immediate donor safety, but repeated donation may affect iron stores, haemoglobin recovery and adverse reactions, requiring active management of cumulative burden. At the same time, collection, processing, inventory, utilisation and haemovigilance data are fragmented across systems and organisations and are not routinely linked. Without clear accountability across this pathway, improving donor value yield is difficult. Vein‐to‐vein databases can provide the technical foundation for linkage, but they do not in themselves constitute a stewardship framework.

We therefore propose Donor Blood Management (DBM), a stewardship framework that builds on these vein‐to‐vein infrastructures to establish accountability, drive improvement, optimise component yield, minimise wastage and ensure appropriate clinical use and haemovigilance. DBM is structured around four interconnected pillars: (i) Collection and processing optimisation, (ii) inventory optimisation, (iii) appropriate utilisation, and (iv) haemovigilance and Improvement.

To measure DBM performance, we introduce Donor‐to‐Recipient Value Yield (DRVY): The proportion of donated units ultimately transfused appropriately. DBM provides the framework for interpreting integrated donor‐to‐recipient data, while DRVY translates that data into a standardised metric of donor value yield.

### 1.1 Donor Advocacy and Ethics

DBM is founded on the principle that every blood donation is a voluntary, altruistic and non‐remunerated gift made for the benefit of others [5, 6]. This creates an ethical obligation for blood services, hospitals, clinicians and regulators to manage donations responsibly and minimise avoidable loss.

While transfusion ethics has traditionally focused on patient safety and appropriate use, DBM extends this frame to preservation of donor value throughout the pathway. Donors have a reasonable expectation that their gift will be managed efficiently and reserved for genuine patient benefit [5, 6, 7, 8]. By promoting stewardship, transparency, and shared accountability, DBM helps protect both donor value and the public trust on which voluntary donation depends [8, 9, 10].

### 1.2 DBM: A Complementary Framework

PBM focuses on the clinical decision: Was this transfusion necessary? Yet this question arrives late. By the time transfusion is considered, the donation has already traversed collection, processing, testing, storage and distribution, each stage where value can be lost. DBM extends stewardship across the entire lifecycle of donated blood, from donation to patient benefit. It is not a replacement for PBM but a complement.

The transfusion pathway is an interconnected system, and the new Substances of Human Origin (SoHO) regulatory framework supports this integrated perspective by requiring Member States to pursue adequate supply, promote donor retention, monitor stocks and encourage optimal use [11]. DBM calls for shared accountability across the chain, measuring success not by inventory alone but by the proportion of donated blood appropriately used for patient benefit.

The need for DBM is urgent. Blood services face ageing donor populations, recruitment challenges and rising demand [12, 13]. In the United States, only about 3% of eligible individuals donate each year [14], while in the United Kingdom, less than 4% of adults are active donors [15]. This reliance on a small donor pool makes stewardship of each donation critically important.

As shown in Figure 1, donor value may be lost at multiple stages—collection failure, processing losses, discard, expiry, inventory imbalance, inappropriate ordering, unnecessary transfusion and incidents—each representing an opportunity for measurement and improvement.

**FIGURE 1:**
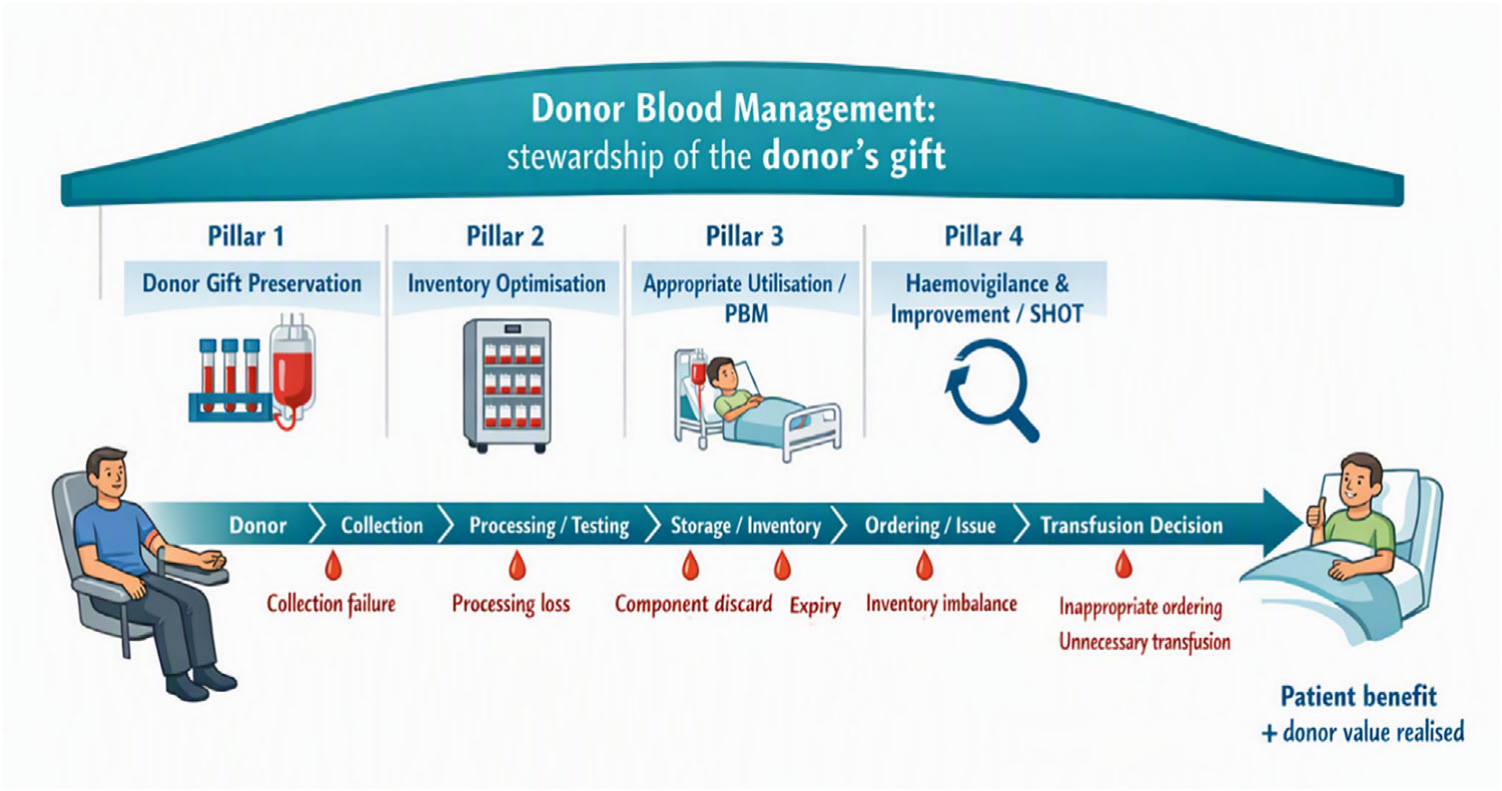
The blood donation pathway. Journey of a blood donation from collection, testing and processing through storage, distribution, clinical decision‐making and transfusion, showing points where donor value may be lost and where vein‐to‐vein data linkage can support measurement and improvement.

DBM is not a radical departure but an organising framework that connects existing efforts—inventory management, appropriate utilisation, haemovigilance—under a coherent, donor‐centred vision.

### 1.3 The Four Pillars of DBM

DBM is built on four pillars that provide the operational structure for the framework. Each pillar addresses a specific stage of the donor‐to‐recipient pathway where donor value can be optimised.

*Pillar 1: Collection and Processing Optimisation*: Led by blood services, balances donor‐centred outcomes—iron status, haemoglobin recovery, adverse reactions, deferral management and donation frequency—with minimising avoidable loss from collection to component production [5, 6, 8, 9, 10]. This is achieved through reducing collection failures, optimising component separation, minimising discard rates and implementing quality assurance via automated processing, real‐time monitoring and standardised quality control.

*Pillar 2: Inventory Optimisation*: Led by blood services and hospitals, focuses on minimising avoidable loss through forecasting, stock rotation, redistribution, expiry prevention and reduced wastage [16]. This is achieved through first‐in‐first‐out stock rotation, near‐expiry redistribution between hospitals, demand forecasting and real‐time inventory tracking. Every expired unit represents a lost opportunity for patient benefit.

*Pillar 3: Appropriate Utilisation*: Led by hospitals and clinicians, ensures that transfusion is evidence‐based and clinically justified [17, 18]. This is achieved through single‐unit transfusion policies, adherence to national guidelines, pre‐transfusion anaemia management and clinician education. PBM asks whether transfusion is necessary; DBM additionally asks whether the donor gift has been used appropriately. The two frameworks converge at the transfusion decision. DBM does not redefine transfusion appropriateness, which remains within PBM.

*Pillar 4: Haemovigilance and Improvement*: Led by haemovigilance programmes, blood services and hospitals, provides feedback through reporting, audit, benchmarking and learning from incidents [19, 20]. Vein‐to‐vein datasets can extend this feedback by connecting donor and product characteristics with recipient outcomes and by identifying losses or variation across the pathway [21, 22, 23, 24]. Examples include SHOT reporting, root cause analysis and benchmarking against national standards.

Putting these pillars into practice requires stakeholders—blood services, hospitals, laboratories, clinicians and haemovigilance programmes—to work across the donor‐to‐recipient pathway rather than within organisational silos (Figure 2). DBM links these responsibilities through shared governance structures, such as Trust Transfusion Committees, and through integrated data systems, including data warehouses and business intelligence tools. Dedicated data analysts are needed to support real‐time monitoring and reporting, while emerging applications of artificial intelligence may support data harmonisation, signal detection and continuous improvement.

**FIGURE 2:**
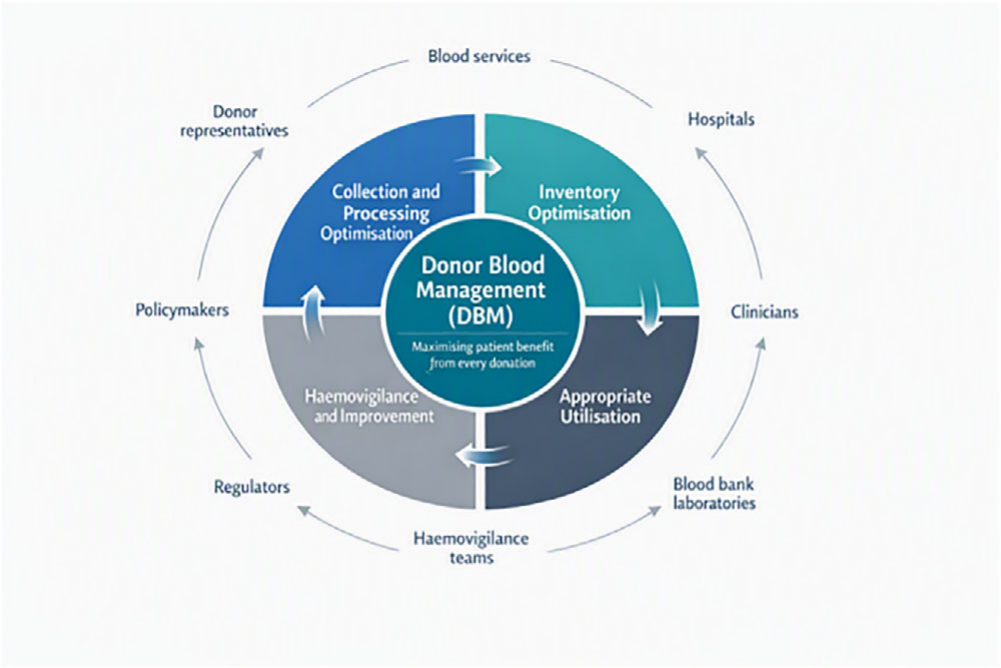
Donor Blood Management framework. The four interconnected pillars—collection and processing optimisation, inventory optimisation, appropriate utilisation and haemovigilance and improvement—supported by vein‐to‐vein data infrastructure, shared governance and quality‐improvement feedback across the donor‐to‐recipient pathway.

### 1.4 DRVY: A Secondary Stewardship Metric

DRVY is proposed as the proportion of donated blood that is ultimately transfused appropriately. It is a system‐level stewardship metric enabled by vein‐to‐vein linkage and should be interpreted alongside established measures of collection loss, component discard, expiry, wastage, issue‐to‐transfusion ratios and clinical appropriateness. DRVY is therefore not a replacement for existing utilisation or wastage measures, but a way to interpret them through a donor‐centred stewardship lens.

$$\begin{array}{ccc} & & \mathrm{DRVY}\left(\%\right)=\left(\mathrm{units}\;\mathrm{or}\;\mathrm{component}\;-\;\mathrm{equivalents}\;\mathrm{appropriately}\right.\\ & & \;\mathrm{transfused}\;÷\;\mathrm{units}\;\mathrm{or}\;\mathrm{component}\;-\;\mathrm{equivalents}\;\mathrm{donated}\;\\ & & \;\left.\mathrm{or}\;\mathrm{produced}\right)\times 100 \end{array}$$

$$\mathrm{DRVL}\left(\%\right)=100-\mathrm{DRVY}\left(\mathrm{donor}\;-\mathrm{to}\;-\mathrm{recipient}\;\mathrm{value}\;\mathrm{loss}\right)$$

For example, if 100 whole‐blood donations are processed into 250 components (for example, red cells, platelets and plasma), and 210 components are transfused appropriately, the DRVY is 84%. The remaining 16% represents donor‐to‐recipient value loss through collection failure, processing loss, expiry, wastage or inappropriate use, and should be analysed across the four DBM pillars.

Because each donation can yield multiple components, DRVY should be calculated separately for red cells, platelets, and plasma. Comparing component‐specific yields identifies where donor value is most frequently lost and where interventions should be prioritised. For apheresis, a double‐ or triple‐dose platelet collection contributes the corresponding number of therapeutic doses to the denominator, not donor attendances.

DRVY does not determine whether a transfusion was appropriate—that judgement remains with PBM. Nor should DRVY be maximised at the expense of donor safety, quality control, contingency stock or equity. A high ratio may be misleading if appropriateness is poorly recorded or if safety losses are excluded without explanation.

DRVY has not yet been measured in practice. Its actual value remains unknown. DRVY depends on data quality and linkage, not data size. With complete vein‐to‐vein linkage, full DRVY can be calculated; with partial linkage, an estimated operational DRVY may be useful if limitations are explicitly reported. The goal is to start measuring with available data while working towards better linkage.

Vein‐to‐vein databases provide the infrastructure for DRVY measurement but have limitations: variable data quality, incomplete capture of outpatient or home transfusions and differing definitions across systems. Any DRVY calculation must therefore report linkage completeness, uncertainty and data limitations.

Importantly, the infrastructure already exists in several vein‐to‐vein systems [21, 22, 23, 24]. The next step is to define consistent stewardship variables and apply them across existing datasets. Haemovigilance programmes such as SHOT demonstrate that avoidable human and system errors remain an important source of preventable loss [20, 25]. Current metrics—units collected, issued and wastage rates—provide only a partial view [16, 26]. DRVY measures what matters: how much of the donor's gift translates into appropriate patient benefit.

However, DRVY should not be interpreted as a simple target to maximise. Some losses are unavoidable (e.g., testing discards, quality control samples). Reporting should distinguish avoidable from unavoidable losses, include safety and equity safeguards, and describe uncertainty from incomplete linkage or clinical data.

DRVY's value lies in providing a transparent summary of system conversion from component production to appropriate patient use. It should remain subordinate to primary DBM outcomes: donor health, donor experience, equitable access and sustainable participation.

Future work should validate DRVY across existing linked systems (e.g., Canadian, Scandinavian), standardise component‐specific denominators, distinguish avoidable from unavoidable losses and use analytics to support improvement. International collaboration and transparent reporting are essential if DRVY is to become a comparable quality‐improvement metric.

Several limitations should be acknowledged. DBM is proposed here as a conceptual stewardship framework and requires operational validation in different blood systems. DRVY will depend on the completeness and quality of linked data, the reliability of appropriateness coding and agreement on component‐specific denominators. Implementation may also vary according to regulatory environment, digital maturity and the division of responsibility between blood services and hospitals.

### 1.5 Call for DBM

DBM reframes blood as a human gift whose value should be protected throughout the donor‐to‐recipient journey [5, 6, 7]. It does not replace PBM, GMP, vein‐to‐vein databases, haemovigilance or existing inventory systems. Rather, it connects them within an explicit stewardship framework that places the donor—their health, burden, experience and accessibility—at the centre of measurement and accountability before, during and after donation.

A blood system is sustainable only if it protects both recipients and the people who make transfusion possible. PBM remains the patient‐centred framework for clinical decision‐making; GMP and quality systems remain the foundation for safe component production. DBM complements them by making the donor a continuing subject of care, accountability and longitudinal learning. PBM asks what care is best for the patient; DBM asks what obligations the blood system has to the donor.

Initial implementation should prioritise donor‐centred outcomes already measurable: iron status, haemoglobin recovery, adverse reactions, deferral and safe return, donor‐reported experience, accessibility, retention and diversity. Future research should determine which outcomes are suitable for benchmarking, how donor‐reported measures should be collected and how trade‐offs between supply resilience and donor burden should be governed.

DBM's value is integrative rather than regulatory. It makes explicit the longitudinal obligations to donors that may be dispersed across standards, operational policies and research programmes, and creates a governance structure through which donor‐health outcomes, donor experience, retention and linked recipient learning can be reviewed together.

By combining ethical accountability, inventory discipline, appropriate utilisation, haemovigilance and linked‐data measurement, DBM offers a practical approach to reducing avoidable loss, strengthening sustainability, maintaining public trust and maximising patient benefit. Vein‐to‐vein infrastructure makes this ambition measurable, and DRVY provides a proposed common outcome through which performance can be interpreted and improved. DRVY should, however, remain subordinate to the primary purpose of DBM: safeguarding donor health and sustaining a fair, trusted and resilient donor community.

The transfusion community should now consider DBM alongside PBM as a core principle of modern practice.

## Author Contributions

A.A. conceptualised the Donor Blood Management framework, drafted the original manuscript, performed the literature review and approved the final version for submission. A.P. reviewed the literature and approved the final version for submission.

## Funding

The authors have nothing to report.

## Ethics Statement

The authors have nothing to report.

## Conflicts of Interest

The authors declare no conflicts of interest.

## Data Availability

Data sharing is not applicable to this article as no new data were created or analysed in this study.
